# An epidemiological study of dementia under the aegis of mental health program, Maharashtra, Pune chapter

**DOI:** 10.4103/0019-5545.64588

**Published:** 2010

**Authors:** D Saldanha, Maj Raghunandan Mani, Kalpana Srivastava, Sunil Goyal, D Bhattacharya

**Affiliations:** Comdt 92 Base Hospital, C/O 56 APO and formerly Prof and HOD Psychiatry AFMC, India; 1 Armed Force Medical College, Pune, India; 2 Graded Spl Psy 151 BH C/O 99 APO, India

**Keywords:** Alzheimer’s dementia, epidemiology of dementia, prevalence of dementia

## Abstract

**Background:**

There has been an exponential growth in the number of elderly population in India. This study aims to determine the prevalence of dementia in an urban center of Pune and to evaluate the corresponding socio-demographic correlates along with psychiatric morbidity in the study sample.

**Materials and Methods:**

The study population in Pune and Kirkee cantonments was selected based on 2001 census data. The number of people over 65 years numbered 6721 and 2145 of them were randomly selected for a door-to-door survey. They were initially administered household questionnaire and then subjected to a screening tool. Each participant underwent a brief mental state examination and data was collected on the basis of a structured proforma. Patients underwent a detailed cognitive profile using subtests from CSI-D (community screening instrument – dementia), which included a Consortium to Establish a Registry for Alzheimer’s Disease (CERAD) word list, word fluency and delayed recall. Information pertaining to socio-demographic factors in participants and caregivers, caregiver-burden and behavioral and psychological symptoms in participants too were collected from the questionnaire. Radio imaging investigation was also carried out to quantify the deficit. Statistical Package for the Social Sciences (SPSS) software was used to compute the results.

**Results:**

Findings revealed that prevalence of dementia in the sample population of elderly aged above 65 years was 4.1%. Socio-demographic factors which conferred a statistically higher risk for dementia were identified to be older age, low socio-economic status, low level of education, presence of family history, whereas, marriage was found to be protective. Burden of care was associated with caring for elderly with dementia with increasing severity of dementia. Patients with dementia performed poorly on cognitive test battery. Social network had a protective effect in respect with severity of dementia. On magnetic resonance imaging (MRI) majority of cases of Alzheimer’s Dementia (AD) and Vascular Dementia (VaD) were noted to have both gray and white matter involvement.

**Conclusion:**

Poor awareness is a key public-health problem. Society plays an important role in the ageing process. The withdrawal of the elderly from the previous societal roles,reduction in all types of interactions i.e. shift of attention from outer world to the inner world, reduction in the power and prestige of the elderly enhance aging process. Aging in Indian culture though a disability is much stressful today in Indian culture as in others.

## INTRODUCTION

**“People don’t grow old, when they stop growing they become old”**

**Anonymous**

Emerson had remarked that "life was unnecessarily too long". The senior citizen is something of a psychological enigma. Venerated by some for his experiential wisdom, tolerated by others for his quaint whims and fancies, ridiculed by many for his doddering senility, he belongs nevertheless to the most rapidly expanding segments of the population in developed societies.

In India, life expectancy at birth has increased by 30 years since independence, and it is higher for women than men. According to the World Health Organization (WHO), India’s population of those aged over 65, which was 40 million in 1997, is set to increase to 108 million by 2025 and 240 million by 2050. This means a several-fold increase in age-related problems such as dementia – a condition characterized by progressively declining memory and intellectual functions. The WHO, which estimates that two out of every three patients with dementia will soon be in developing countries, appears to be a virtual dementia epidemic in India and the urgent need to prepare to face it.[1] The rates of prevalence of dementia in India are quoted to be lower than in Western countries; however, it is still going to be a major problem unless an effective intervention is planned. Studies have suggested that the prevalence of dementia may be considerably lower in developing than in developed parts of the world.[2] India’s population rising to be approximately more than one billion, about 60 million are over 65 and at least 2.5 million have dementia. It is estimated that by year 2020, eight million individuals in India will be affected by dementia.[3] Alzheimer’s disease is one of the most disabling and burdensome health conditions worldwide, with an estimated 24·3 million people having dementia today, with 4·6 million new cases of dementia every year. Most people with dementia live in developing countries and numbers forecast an increase by 100% between 2001 and 2040.[4,5]

The burden of disease is shared and reduced by increasing the awareness in of the disease *per se*. The diseases of old age indeed constitute a major challenge for contemporary biomedical research.

Difference in rates of prevalence of dementia in Indian setting may be ascribed to different locations of the study sample size and the methodology followed by researchers. In the Indian setting, studies are carried out at cross sectional and zonal basis. A study on the prevalence of dementia in a rural population, conducted on 750 elderly (60 years of age and older) persons of a community on the outskirts of Chennai city in South India, using the Geriatric Mental State schedule (GMS), noted prevalence rate to be 3.5%; the percentage increasing with age. These rural prevalence estimates were higher than in urban settings.[6]

Providing appropriate help from medical and social welfare services may be hindered if people with dementia and their relatives are unaware of the help available, or if they perceive it as inappropriate for them. This lack of awareness hinders the analysis of prevalence of dementia research, well disseminated, can play an important role in increasing awareness at all levels of society.[7,8]

Epidemiological reports about dementia in elderly people from developing countries, including India, are sparse.[9,10] Most of these studies are either part of general population studies, community studies or based on hospitalized patients. Very scant data exists about the mental health status of elderly living in community.

A factor hindering the analysis of prevalence of dementia in developing nations is the lack of dementia awareness. Research, well disseminated, can play an important role in increasing awareness at all levels of society. Health care systems existing in developing countries will need to be kept updated on the changing demands and it is critical to prepare oneself for this upcoming explosion in the number of mentally ill elderly persons.[11]

Studies on resources, needs and outcomes in community-based and care of the elderly would help in evolution of strategies on evidence based practices in geriatric mental health care. Hence the present study was undertaken to evaluate prevalence of dementia and other psychiatric co-morbidities in an urban center of Pune and to understand the level of awareness among the carers of these cases.

## MATERIALS AND METHODS

This study was carried out in Pune and Kirkee cantonments, Maharashtra. The study population was identified from existing registry, held by cantonment authorities. Of the whole population, those aged more than 65 years totaled **to** 6721 residents. The total period of study extended from July 2005 – September 2007.

Those aged more than 65 years, residents of catchment’s area and who were willing to participate in the study, were included in the study. Subjects with physical disability or with any medical illness rendering individual incapacitated to interact meaningfully were excluded from the study.

A sample size for study comprised of 2145 elderly population of the cantonment. After the cases were selected, by random sampling, they were identified as per the research protocol by door-to-door knocking to identify the resident elderly. Of those selected, 12 died and 14 refused consent/relocated/ did not meet the inclusion criteria. Hence, consent was taken from 2119 residents of the community who were examined for dementia and other co-morbidities.

A predetermined questionnaire, in vernacular (Marathi) adaptation was administered and information was collected by trained research assistants in a face to face interview in the elderly identified population.

A brief mental state examination was carried out in all subjects. Mini Mental State Examination (MMSE) was used as a screening tool. Those found to have score of 23 or below were subjected to further tests to obtain cognitive profile.

Cognitive screening instrument for dementia included a ‘cognitive test component’ of the 10/66 research groups instrument – community screening instrument for Dementia (CSI-D), which included a ‘Consortium to Establish a Registry of Alzheimer’s Disease (CERAD) 10 word list (CWL), an ‘animal naming verbal fluency’, and the ‘delayed recall of word’ test.

Information on household structure, socio-demographic factors, caregiver information were collected using appropriate questionnaires viz. ‘household questionnaire’, ‘socio-demographic and risk factor questionnaire’ ‘participant version’ or ‘informant version’; ‘care-giver burden’ including informant module, background module and care module. Informant module contains a section of CSI-D and NPI-Q (neuro-psychiatric Inventory – Questionnaire), which details information about behavioral and psychological symptoms. The ‘background module’ contains information on the socio-demographic background of the care giver and was administered to all the informants. The ‘care module’ which was the last one, was administered only if the older person had some care need as ‘identified subjectively by the caregiver’. The data so collected in a structured manner was entered on a response code booklet. Diagnosis was made on the basis of ICD-10 Diagnostic Research Criteria.[12] The data recorded on the questionnaire was entered into SPSS software and analyzed using the software.

An initial cross sectional survey helped obtain the demographics of the population and study the prevalence of dementia. As reliable information pertaining to some variables being studied in association with dementia, was not available for the entire sample, hence further analysis was based on the pattern of population based case control study where the set of data pertaining to the cases were taken along with age and gender matched controls from the community surveyed.

## RESULTS

**Figure 1:**
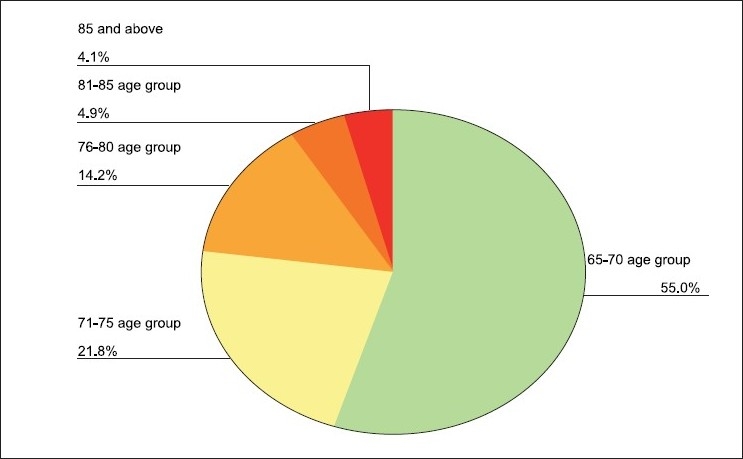
Age distribution of the sample population

**Figure 2:**
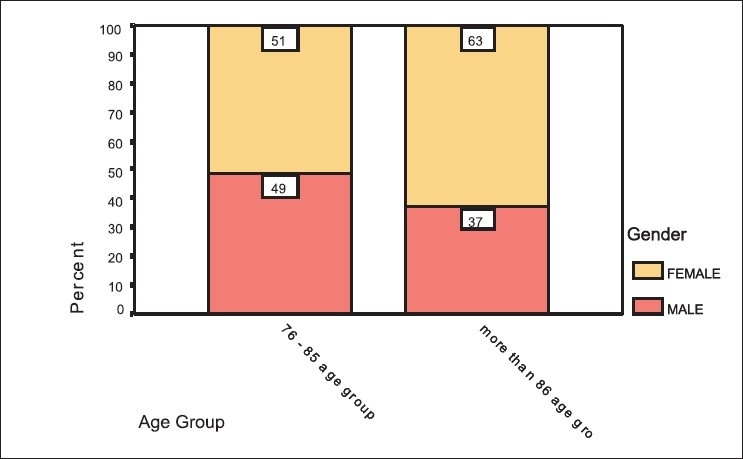
Gender distribution in various age group

The mean age of the sample was 71.94 ± 6.58 years [Table 1, Figure 1]. Demographic data of the sample shows that there is a uneven gender distribution of the population sampled with a female predominance who constituted 60.5 % of 2119 studied while male constituted 39.5 % of the whole [Table 2, Figure 2]. The proportion of females in age group was seen to increase with increasing age and when cross tabulated with age group below 85 and above 86 it was seen that the proportion of females increased from 51% in the former to 63% in the latter group, which was statistically significant (*P*< 0.001).

**Table 1:** Mean age of the sample population

Total	**2119**
Mean	**71.94**
Std. Deviation	6.58
Minimum	**65**
Maximum	106

**Table 2:** Gender distribution in various age groups

			Gender		Total
			Male	Female	
**Age Group**	65 ‐ 75 age group	Number	605	1015	**1620**
		% within Age Group	37.3%	62.7%	100.0%
		% within Gender	72.3%	79.2%	76.5%
	76 ‐ 85 age group	Number	196	206	**402**
		% within Age Group	48.8%	51.2%	100.0%
		% within Gender	23.4%	16.1%	19.0%
	More than 86 age group	Number	36	61	**97**
		% within Age Group	37.1%	62.9%	100.0%
		% within Gender	4.3%	4.8%	4.6%
Total		Number	837	1282	**2119**
		% within Age Group	39.5%	60.5%	100.0%
		% within Gender	100.0%	100.0%	100.0%
Chi Square<0.001					

with OR = 1.16 , 95% CI = 0.7 – 1.04, *P* = 0.49.

The sample population had higher percentage of people who were illiterate [Table 3]. Figures pertaining to education showed that the percentage of people with dementia was more in the ‘Illiterate’ group – 4.1%, and it fell in those with higher levels of education, ‘middle school or lower’ to 3.9% and was 0.5% in those with ‘secondary school’ level of education. Paradoxically, those with ‘graduation or higher’ education had the maximum proportion of elderly with dementia, which was statistically significant (*P*< 0.09).

**Table 3:** Education status and dementia cross-tabulated

Prevalence of Dementia and Educational status			
Educational status	Dementia absent (Number)	Dementia present (Number)	Total (Number)
Illiterate	1293	55	1348
Middle school or lower	439	18	457
Secondary school	197	1	198
Graduation or higher	65	3	68
Total	1994	77	2071
Chi-Square Tests	Value	df	Sig. (2-sided)
Pearson Chi-Square	6.357	3	0.09

*P*<0.09

Majority of the persons studied were married - 48.3% and only 1% of the sample groups were from the ‘divorced or separated’ group [Table 4]. Marriage had a significant difference on prevalence of dementia those who were married represented the corresponding age groups in greater proportion than those who were ‘never married’, in either gender [*P*<.05 Table 4]. Within group analysis of the marital status showed that there were higher number of people with dementia among the ‘never married’ group (25%), followed by the ‘divorced/separated’ group (5%), the ‘widow/ widower’ group, (3.4%) and least in those ‘married and co-habiting’ (2.2%). The risk of dementia in those ‘never married’ was found to be 11 times higher than compared to those who were ‘ever married’. OR = 11, (95% CI 6.2 – 20.4, *P*<0.001).

**Table 4:** Marital status and dementia cross tabulated

	Dementia		Total
	Absent	present	
Marital status			
Never married	66	22	88
Married/ Co-Habiting	980	22	1002
Widowed	931	33	964
Divorced/ separated	19	1	20
Total	1996	78	2074
Chi-Square Tests	Value	df	Sig. (2-sided)
Pearson Chi-Square	116.849	3	0.001

OR = 11, (95% CI 6.2 − 20.4, *P*<0.001).

Analyzing socio-economic status as a factor affecting prevalence of dementia revealed that in those from lower status it comes to occupy 60%, 34.8% in ‘high middle’ group and 25% in those from ‘high’ group. This trend was statistically significant [*P*<0.001, Table 5]. When the data was compressed into two groups, ‘high to middle status’ and ‘low middle to low status’, the risk was found to be more than three-fold higher in those from the lower socio-economic strata. (Odds ratio 3.95, 95% CI 1.99 – 7.89, *P*<0.001).

**Table 5:** Socio-economic status and cross tabulation with dementia

Kuppuswamy’s Socioeconomic Index (SES)	Prevalence of Dementia		
	
	Dementia Absent	Dementia Present	Dementia Total
High SES	3	1	4
High middle SES	15	8	23
Middle SES	34	15	49
Low middle SES	24	47	71
Low SES	10	15	25
Total	86	86	172

Odds Ratio 3.95, 95% CI 1.99 – 7.89, *P*<0.001).

The analyzing effect of family history showed that in those with a history of dementia 85.7% (6/7) had dementia compared to the group without family history, where 3.5% (72/2072) had dementia. The odds ratio for dementia was worked out to be 24 times higher in the group with family history (OR 24.67, 95% CI 6 – 87, *P*<0.001)(Fishers exact method) [Table 6].

**Table 6:** Dementia and family history

Family history of dementia	Prevalence of dementia		
	
	Dementia absent	Dementia Present	Total
Negative history	2000	72	2072
Positive history	1	6	7
Total	2001	78	2079
Chi-Square Tests	Value	df	Asymp. Sig. (2-sided)
Pearson Chi-Square	130.665	1	<0.001

(OR 24.67, 95% CI 6 – 87, *P*<0.001)

Reflection of social network and dementia showed that worsening of network was associated with increased proportion of elderly with dementia, increasing from 3.8% of the elderly with dementia with good social network, to 62.5% elderly with dementia in average network and 85% of elderly with dementia with poor social network [Table 7 *P*<0.001].

**Table 7:** Social–network and dementia

Social network	Prevalence of dementia		
	
	Dementia absent	Dementia present	Dementia total
Good social network	50	2	52
Average social network	30	50	80
Poor social network	6	34	40
Total	86	86	172

*P*<0.001

Prevalence of dementia revealed [Table 8] proportion of people with dementia is increased with older age groups and prevalence of dementia constitutes 2.8% in those between 65-70 years age group and increases to 18.4% in the oldest old (aged 86 and above). This linear association is noted to be highly significant (*P*<0.001).

**Table 8:** Prevalence of dementia across age groups

Age group		Dementia		Total
		Absent	Present	
65-70 age group	Number	1129	32	1161
	% within age	97.2%	2.8%	100.0%
	% within dementia	55.5%	37.2%	54.8%
71-75 age group	Number	449	10	459
	% within age	97.8%	2.2%	100.0%
	% within dementia	22.1%	11.6%	21.7%
76-80 age group	Number	281	18	299
	% within age	94.0%	6.0%	100.0%
	% within dementia	13.8%	20.9%	14.1%
81-85 age group	Number	93	10	103
	% within age	90.3%	9.7%	100.0%
	% within dementia	4.6%	11.6%	4.9%
86 and above	Number	71	16	87
	% within age	81.6%	18.4%	100.0%
	% within dementia	3.5%	18.6%	4.1%
Age data not available	Number	10		10
	% within age	100.0%		100.0%
	% within dementia	.5%		.5%
	Number	2033	86	2119
	% within age	95.9%	4.1%	100.0%
	% within dementia	100.0%	100.0%	100.0%
Value		df	Asymp. Sig. (2-sided)	
66.941		5	<0.001	
43.594		1	<0.001	

MMSE was used to screen patients of dementia showed that of the total 2119 surveyed, 129 had scored below the predetermined score of 23. Of those screened positive with MMSE, 86 were clinically diagnosed to be having dementia (ICD 10, DCR). Those who had dementia had lower MMSE scores than those who did not (17.88 versus 22.06 respectively *P*<.005), [Table 9]. The mean MMSE was tabulated with the clinical severity of dementia and it was found to be 22.06 in those without dementia and shows a trend of decreasing scores with increasing severity of dementia [*P*<.005 ,Table 10]. Recall of a word list (from CERAD), ‘word fluency test’ and ‘delayed recall’ too showed a decreasing scores with increasing severity of dementia [Table 11, *P*<0.001].The psychiatric symptoms were found to be significantly higher in the population with dementia than in those without dementia [Table 13 and 13A].

**Table 9:** MMSE scores and prevalence of dementia

	ICD 10 (DCR)	
	Dementiapresent	Dementia absent
Mean MMSE score	86	43
	17.88	22.06
	(SD 4.93)	(SD 0.35)

**Table 10:** MMSE and severity of dementia

	Mean	N	Std. deviation
No dementia	22.06	43	0.35
Mild dementia	21.10	42	1.57
Moderate dementia	16.00	39	3.87
Severe dementia	5.60	5	4.93
Total within dementia group	17.88	86	3.02
Total number		129	

*P*>0.001

**Table 11:** Cognitive profile recall of_CERAD word list

	Mean	N	Std. deviation
No dementia	17.68	31	2.59
Depression	15.50	8	3.12
MCI	14.00	4	.00
Mild dementia	9.90	42	2.99
Moderate dementia	7.87	39	1.84
Severe dementia	5.00	5	2.51
Total	11.44	129	4.80

ANOVA<0.001

**Table 12:** Cognitive profile; word fluency

	Mean	N	Std. deviation
No dementia	16.06	31	1.04
MCI	16.75	4	1.26
Depression	16.00	8	.76
Mild dementia	8.24	42	3.93
Moderate dementia	1.15	39	2.01
Severe dementia	3.33	5	.82
Total	9.99	129	5.27

ANOVA Table<0.001

**Figure 3:**
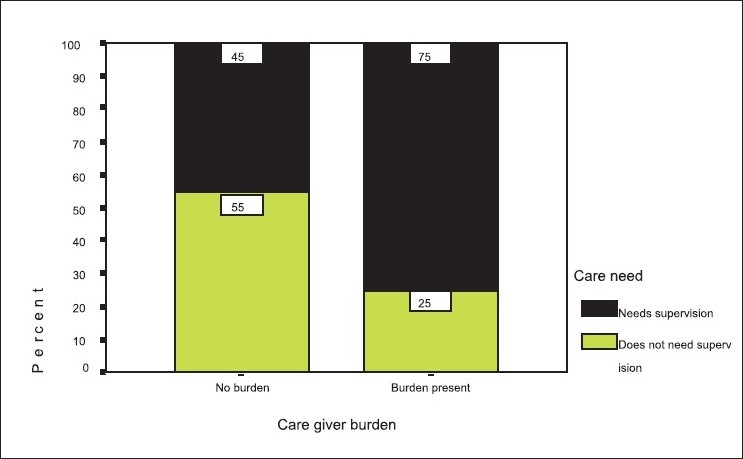
Caregiver burden versus care needed by the elderly

Analyzing further, the severity of dementia across the grades of projected care burden, showed a proportionately linear trend [*P*<0.001, Figure 3]. The proportion of caregivers experiencing burden increases when caring for the elderly with dementia than when caring for those without. [Table 14 *P*<0.001 Figure 3] MRI findings also revealed that within the subgroups of dementia, majority of cases of AD and VaD were noted to have both gray and white matter involvement. Within the AD subgroup only cortical gray matter involvement was seen in 7.8% cases compared to 74.5% cases that had a mixed gray and white matter involvement. Among those with Parkinson’s subtype of dementia, a majority were seen to have sub cortical gray matter involvement [Table 15].

**Figure 4:**
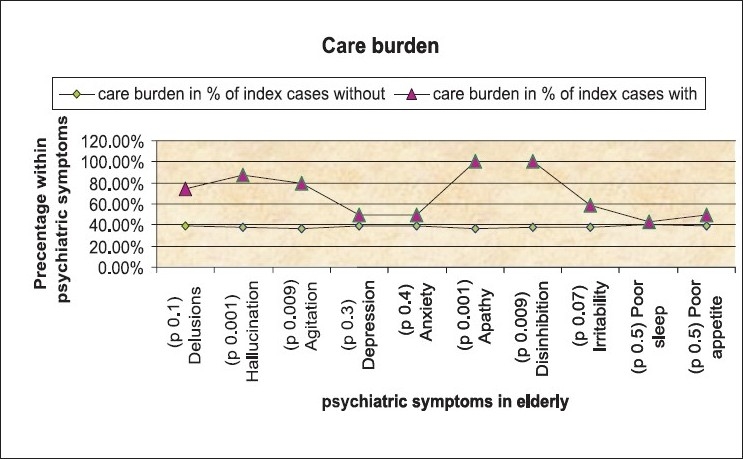
Care burden and psychiatric symptoms

## DISCUSSION

This study is comprehensive in its aim and evaluates not only the prevalence of dementia but also the burden of care associated with dementia. The mean age of the sample is 71.94 years [Table 1 and Figure 1] where in 85 and above comprise only 4.1% and 14.2% fall in the 76 to 80 years of age. In India, a steady demographic transition with increase in the elderly population is changing the population structure from a wide based pyramid to a barrel- shaped form.[13] It is interesting to note that the proportion of females in age group was seen to increase with increasing age. This contrasts with the increased male to female ratio in the younger group, but confirms to the data that women outlive men and consequently, as quoted by WHO, in the old age the ratio reverses to almost 2. This contrasts with the increased male to female ratio in the younger group, but confirms to the data that women outlive men and consequently, as quoted by WHO, in the old age the ratio reverses to almost 2:1.[14] Partly survival of female species is quoted to be one of the reasons. Demographic profile shows increased proportion of females compared to males, which could be reflecting a trend of increased survival in females with increasing age [Table 2 and Figure 2].[15]

Educational status had the protective effect [Table 3].There was significant difference in the educational standards and evidence of dementia. The effect of education on the cognitive decline can be explained using the ‘cognitive reserve hypothesis’ with a positive correlation that a low level of education is related to an increased incidence of dementia. In this study, the percentage of people with dementia was more in the ‘Illiterate’ group though, paradoxically, those with ‘graduation or higher’ education had the maximum proportion of elderly with dementia, which was statistically significant (*P*<0.09). Further analysis did reveal that those with higher education were in the older age group, with more severe grade of dementia. This further strengthened the ‘cognitive reserve’ hypothesis that those with higher educational attainment take more time to deteriorate than their counterparts with lower educational status; the same was noted in other findings carried out in urban settings.[16–18]

Marital status has been known to be a predictor of longevity. In our study, we found that number of married represented the corresponding age groups in greater proportion probably reflecting the longevity. Further, it was seen that the risk of dementia was higher in those who were either not married or separated [Table 4]. These findings have been consistent with other studies too, where they have found that the prevalence of dementia was highest in ‘unmarried’ group’.[19] The socio-economic status (SES) is an important determinant of health and lower socio-economic status has been found to be associated with higher prevalence of dementia [Table 5] Socio-economic status also influences the accessibility, affordability, acceptability and actual utilization of various available health facilities. It could be argued that cognitively challenged people have less economic opportunity and reserves, but the converse could hold true, that the lower social and economic integration might lead to a worsening in the cognitive decline.[20] A higher risk of dementia with decrease in SES was found in our study [Table 5]; odds ratio was also significant.

People with a family history of the disease are at higher than average risk for Alzheimer’s disease, a finding which the researchers are relating to important genetic factors, notably the ApoE4 gene, but the findings are mixed.[20] In this study we found a higher risk in those with family history. Probably larger data is required to generalize the findings. The study also indicates positive family history as a risk factor.

One of the many factors which are affected in the elderly, as they grow older, is ‘communication’. Studies have found that a decrease in duration, quality or intensity of activity results in a stronger cognitive decline.[21] Decreased vision and/or hearing acuity, restricted mobility often result in poor communication and psychosocial functioning, social dissatisfaction and poor social contact which affect their physical and mental well-being.[22] In our study it was found that worsening of social network, restriction in mobility and hearing loss were associated independently with increased proportion of elderly with dementia [Table 7].

In the present study, the prevalence of dementia in the community was seen to be 4.1% [Table 8].The percentage of those with dementia showed an increase from 2.8% in the age group of 65 – 70 years, to 18.4% in the oldest old living in the community. The risk of dementia increases more than five fold in the old oldest old. (Odds Ratio 5.52 95% CI 2.8 – 10.1, *P*<0.001) [Table 8].

Prevalence of dementia varies from one study to another depending on the catchment area and the demographic profile of the study population. Previous studies in India focusing on the community have found a prevalence rates of 3.39 – 3.5 in rural community[6,10] and 2.4 – 2.7 from urban community.[18,22] In the present study the prevalence of dementia in the community was see to be 4.1%. This urban divide could be explained by the awareness about the problem as in the community surveys from the rural areas, the informant who are the source of information and are often not aware of the nature of illness.

The prevalence rates of dementia in our study showed a linear trend of increasing proportion which was seen to be highly significant; the rate being comparable to the data from other community studies from India[10,18] Analysis put the risk of dementia to a fivefold increase in the oldest old. This trend of increasing prevalence of dementia with age is evident in the present study. Studies have found that gender has a role to play in dementia with risk being higher in women but the results are equivocal.[23] Of those screened positive with MMSE, 86 were clinically diagnosed to be having dementia (ICD 10, DCR). Those who had dementia had lower MMSE scores than those who did not (17.88 versus 22.06 respectively *p*<.005), [Table 9]. The decrease in performance is noted on all the parameters of cognitive domain [Tables 10‐12].

Hence, it was noticed that with increasing severity of clinically diagnosed dementia there was an attendant fall in scores too . This trend was analyzed using ANOVA between groups and was found to be highly significant (*P*<0.001) This finding was lower compared to another similar study, from Brazil, where the mean was found to be 11 ± 4.1 for mild and 7.4 ± 2.6 for moderate dementia.[23] Our findings find the decreased performance related with age.

In a current study targeting cognitive status and behavioral problems in elderly it was found that anxiety, depression, irritability, and agitation/aggression were the most commonly observed behaviors. Hallucinations and delusions were associated with the highest levels of severity and distress; however, they occurred rarely [Table 13] Analysis of psychological symptoms showed that the commonest symptom was irritability (15.1%) followed by depression (5.8%), agitation (5.8%), poor sleep (5.2%), seeing things others cannot (4.7%), unconcern (4.1%), anxiety (3.5%), disinhibition (2.9%), suspiciousness (2.3%) and poor appetite (2.3%). Hence, this study showed that there is a higher prevalence of psychological and behavioral symptoms associated in elderly with dementia [Table 13 A].

**Table 13:** Psychiatric symptoms (NPI –Q)

		Delusions	Hallucination	Agitation	Depression	Anxiety
Not Present	N=172	168 (97.7%)	164	162	162	166 (96.5%)
			(95.3%)	(94.2%)	(94.2%)	
Present		4	8	10	10	6
		(2.3%)	(4.7%)	(5.8%)	(5.8%)	(3.5%)
Present	N=86	4	7	8	6	6
		(2.3%)	(8.1%)	(9.3%)	(7%)	(7%)
Pearson’s Chi-square ‘p’		0.06	0.03	0.05	0.37	0.014

**Table 13 A:** (Continued): Psychiatric symptoms (NPI –Q)

		Apathy	Disinhibition	Irritability	Poor sleep	Poor appetite
Not Present	N=172	165 (95.4%)	167	155	163	168 (97.7%)
			(97.1%)	(90.1%)	(94.8%)	
Present		7	5	17	9	4
		(4.1%)	(2.9%)	(9.9%)	(5.2%)	(2.3%)
Present	N=86	7	5	13	5	4
		(8.1%)	(5.8%)	(15.1%)	(5.8%)	(4.7%)
Pearson’s Chi-square ‘p’		0.007	0.02	0.01	0.23	0.06

Burden of care also showed significant association with severity of dementia [Table 14, Figures 3 and 4]. Looking after frail elderly for a long time seems to be an important factor related to caregivers’ developing psychological feelings of heavy burden. Analyzing our group, it was found that caregiver-burden was found to be significantly higher in those caring for the elderly with dementia, with increased projection of burden with need for supervision. The results indicate that caregivers of impaired elderly with behavioral disturbances were more likely to feel a ‘heavier burden.’ Caregiver-burden could be reduced by recommending a more ready access to respite services in emergencies.[24]

Neuroimaging has its relevance in aiding diagnosis in the borderline cases with clinical confounding and also in refining the diagnosis, assessing prognosis and in some cases monitoring the treatment. On the MRI sections, vascular dementia is invariably associated with an increased prevalence of infarcts and more extensive white matter change and frontal lobe atrophy is more prominent in frontotemporal dementias.[25] Tabulating the findings of MRI, it was found that majority of the cases of Alzheimer’s dementia had both gray and white matter involvement (74.5%). Predominantly cortical gray matter involvement was seen in 8.1% cases of Alzheimer’s dementia and 7.7% cases of vascular dementia [Table 15].

**Table 14:** Caregiver burden and increasing severity of dementia

			No dementia	Mild dementia	Moderate dementia	Severe dementia	Total
Zarits caregiver	No burden	Number	82	16	6		104
burden		% within burden	78.8%	15.4%	5.8%		100.0%
		% within dementia	95.3%	38.1%	15.4%		60.5%
	Mild burden	Number	3	26	14	1	44
		% within burden	6.8%	59.1%	31.8%	2.3%	100.0%
		% within dementia	3.5%	61.9%	35.9%	20.0%	25.6%
	Moderate burden	Number	1		18		19
		% within burden	5.3%		94.7%		100.0%
		% within dementia	1.2%		46.2%		11.0%
	Severe burden	Number			1	4	5
		% within burden			20.0%	80.0%	100.0%
		% within dementia			2.6%	80.0%	2.9%
Total		Number	86	42	39	5	172
		% within burden	50.0%	24.4%	22.7%	2.9%	100.0%
		% within dementia	100.0%	100.0%	100.0%	100.0%	100.0%
Chi-Square Tests		df			Asymp. Sig. (2-sided)		
Pearson Chi-Square		9			<0.001		

The proportion of caregivers experiencing burden increase in severity with increasing severity of dementia

**Table 15:** MRI findings in dementia: Cortical / subcortical, gray/white matter

	MRI findings					
Types of Dementia	Cortical gray matter involvement N (%)	WM involvement only N (%)	WM and gray matter involvement N (%)	Subcortical gray matter involvement N (%)	Atrophy N (%)	Total N (%)
AD	4(6.5%)	8(12.9%)	48 (74.5%)	1(1.6%)	1(1.6%)	100.0%
VaD	1(7.7%)	2(15.4)	10(76.9)			13
Fronto temporal		1(25%)	3(75%)			4(100%)
Parkinsons type		1(14.3%)	2(28.6%)	4(57.1%)		100.0%
Total						
% within dementia	5.8%	14 %	73.3%	5.8%	1.2%	100.0%

AD: Alzheimer disease; VaD: Vascular dementia

## CONCLUSION

This study addresses the challenges in assessment of dementia. A change in the care for dementia in developing countries can be brought about only by bringing a change in the way dementia is addressed in the community. We need to care for the elderly in a way that they are assured of their happiness and security.

***As Albert Einstein had said that "The intuitive mind is a sacred gift, and the rational mind its faithful servant. We have created a society that honors the servant and has forgotten the gift"***.

Poor awareness is a key public-health problem which has important consequences for the care givers and other members of the family. The burden of care on family remains to be a matter of concern along with the patients of dementia. Future direction should address the follow up of the cases of dementia to understand the progression of the disease and its incidence, apart from strategies for caregivers. The findings have shown clearly the enormous economic and health impact of caring for dementia. These studies will play a crucial role in forming policy on the development of services for people with dementia, and for older people and their families in general. The project aims to generate from the evidence gathered practicable intervention strategies that can be applied and evaluated (in a later research phase) within the same population.

## References

[1] Ferri CP, Prince M, Brayne C, Brodaty H, Fratiglioni L, Ganguli M (2005). Global prevalence of dementia: A Delphi consensus study. Lancet.

[2] Patel V, Prince M (2001). Ageing and mental health in a developing country: Who cares? Qualitative studies from Goa.

[3] Prince M (2000). Methodological issues in population-based research into dementia in developing countries.A position paper from the 10/66 Dementia Research Group. Int J Geriatr Psychiatry.

[4] Kumar AK (2003). Frontline. Indian Natl Mag.

[5] Dias A, Samuel R, Patel V, Prince M, Parameshwaran R, Krishnamoorthy ES (2004). The impact associated with caring for a person with dementia: A report from the 10/66 Dementia Research Group’s Indian network. Int J Geriatr Psychiatry.

[6] Rajkumar S, Kumar S, Thara R (1998). Thara prevalence of dementia in a rural setting: A report from India. Int J Geriatr Psychiatry.

[7] Bowling AP (1989). Contact with general practitioners and differences in health status among people over 85 years. J R Coll Gen Pract.

[8] Liu HC, Chou P, Lin KN, Wang SJ, Fuh JL, Lin HC (1994). Assessing cognitive abilities and dementia in a predominantly illiterate population of older individuals in Kinmen. Psychol Med.

[9] Matthews FE, Dening T (2002). UK Medical Research Council Cognitive Function and Ageing Study. Prevalence of dementia in institutional care. Lancet.

[10] Shaji S, Promodu K, Abraham T, Roy KJ, Verghese A (1996). An epidemiological study of dementia in a rural community in Kerala. Br J Psychiatry.

[11] Bruce ML, McAvay GJ, Raue PJ, Brown EL, Meyers BS, Keohane DJ (2002). Major depression in elderly home health care patients. Am J Psychiatry.

[12] (2004). WHO Geneva. The ICD-10 classification of mental and behavioral disorders: Diagnostic Criteria for Research, 1993.

[13] Chanana HB, Talwar PP (1987). Ageing in India: Its socio-economic and health implications. Asia Pac Pop J.

[14] (2000). WHO: Annual Report of the Director- Health situation, analysis, Geneva.

[15] Hebert LE, Scherr PA, McCann JJ, Beckett LA, Evans DA (2001). Is the risk of developing Alzheimer’s disease greater for women than for men?. Am J Epidemiol.

[16] Qiu C, Bäckman L, Winblad B, Agüero-Torres H, Fratiglioni L (2001). The influence of education on clinically diagnosed dementia incidence and mortality data from the Kungsholmen Project. Arch Neurol.

[17] Sachdev P (2000). Is it time to retire the term "Dementia"?. J Neuropsychiatry Clin Neurosci.

[18] Vas CJ, Pinto C, Panikker D, Noronha S, Deshpande N, Kulkarni L (2001). Prevalence of dementia in an urban Indian population. Int Psychogeriatr.

[19] Kristjansson B, Helliwell B, Forbes WF, Hill GB (1999). Marital status, dementia and institutional residence among elderly Canadians: The Canadian study of health and aging. Chronic Dis Can.

[20] Olarte L, Schupf N, Lee JH, Tang MX, Santana V, Williamson J (2006). Apolipoprotein E epsilon4 and age at onset of sporadic and familial Alzheimer disease in Caribbean Hispanics. Arch Neurol.

[21] van Gelder BM, Tijhuis MA, Kalmijn S, Giampaoli S, Nissinen A, Kromhout D (2004). Physical activity in relation to cognitive decline in elderly men, The FINE Study. Neurology.

[22] de Silva HA, Gunatilake SB, Smith AD (2002). Prevalence of dementia in a semi-urban population in Sri Lanka: Report from a regional survey. Int J Geriatr Psychiatry.

[23] Alberca R, Montes-Latorre E, Gil-Néciga E, Mir-Rivera P, Lozano-San Martín P (2002). Alzheimer’s disease and women. Rev Neurol.

[24] Kumamoto K, Arai Y, Ueda T, Washio M (2004). Cross-validation of the short version of the Japanese version of the Zarit Caregiver Burden Interview (J-ZBI_8). Nippon Ronen Igakkai Zasshi.

[25] Cochrane JM (2006). Neuroimaging for dementia and Alzheimer’s disease. Radiol Rounds.

